# Palinopsia Following Acute Unilateral Partial Vestibular Deafferentation: A Case Report

**DOI:** 10.3389/fneur.2018.00773

**Published:** 2018-09-21

**Authors:** Caterina Stafuzza, Theodore Landis, Jean-Philippe Guyot

**Affiliations:** ^1^Service of Oto-Rhino-Laryngology, Head and Neck Surgery, Department of Clinical Neurosciences, University Hospital of Geneva, Geneva, Switzerland; ^2^Private Practice in Neurology, Geneva, Switzerland

**Keywords:** palinopsia, vestibular deafferentation, room tilt illusion, canalar-otolithic interactions, visual-vestibular interactions

## Abstract

Palinopsia is defined as the persistence or reappearance of images after cessation of the visual stimulus. One patient presented episodes of palinopsia after the functional loss of the 3 semicircular canals of the right ear while the otolithic function was preserved. None of classical causes was identified in this patient, intoxications, brain tumors, migraines, psychiatric disorders, etc. For a movement to be perceived as a single event, central processes of temporal integration are necessary to correct the shift between the rapid vestibular information, and the slow visual information. However, it has been shown on animal models that vestibular inputs are slower than normal in case of peripheral deafferentation limited to the canalar function with preservation of the otolithic function, which is the case in this patient. Therefore, we hypothesize that episodes of palinopsia he presents result from the fact that temporal integration processes do not take into account the slower than normal vestibular information due to the peripheral disorder and continue to slow it down. Thus, the patient keeps the visual image in memory until the late arrival of the vestibular information.

## Introduction

Palinopsia is defined as “the persistence or recurrence of visual images after the stimulus has been removed” (1–3). This phenomenon must be distinguished from the common physiological afterimages generated by a bright visual stimulus, where the mechanism is clearly based on the intensity and the contrast of the stimulus, the time of fixation and the retinal adaptation state (4).

Palinopsia are categorized first according to the delay between the stimulus and the onset of afterimages. In the “immediate type” the delay is null while in the “delayed type” it can vary from a few minutes to hours (4). They are also classified according to the description reported by patients. The so called “hallucinatory palinopsia” are represented by long-lasting, isochromatic and high resolution afterimages that are not influenced by light or motion, while “illusory palinopsia” include unformed, indistinct, and low-resolution afterimages that are affected by environmental conditions. It is generally accepted that the former are linked to dysfunctional visual memory usually caused by posterior cortical lesions or seizures, and the latter to dysfunction in visual perception most frequently caused by migraines, prescription, or illicit drugs, and head trauma (3, 5).

We report on a patient who developed an “immediate hallucinatory palinopsia” after a right vestibular loss following the surgical removal of a giant and aggressive cholesteatoma invading part of the inner ear. To the best of our knowledge a link between palinopsia and peripheral vestibular disorder has never been reported in the literature.

A written informed consent was obtained from the patient for the publication of this case report.

## Case report

A 47 years-old painter, without previous health problem, underwent a petro-mastoidectomy in December 2012 for a voluminous cholesteatoma extending to the inner ear on the right side. The cholesteatoma had exposed the meninges of the middle cerebral fossa, the geniculate ganglion and the horizontal portion of the facial nerve, and it invaded the lateral semicircular canal. The malleus, and the incus body were normal, but the long incus process was necrotic. The stapes was in place, with a preserved mobility. A canal wall down procedure was performed (6). After the diseased tissues were removed the lateral semicircular canal was plugged with pieces of perichondrium covered with bone wax, and the ossicular chain reconstructed placing a minor columella between the tympanic membrane and stapes head (7).

Immediately after surgery the patient noticed a total hearing loss on the operated side, and experienced the typical static and dynamic symptoms consecutive to an acute unilateral vestibular deafferentiation, i.e., a deviation toward the right and a blurred vision while walking. His description of the dynamic vision alteration was particular in that he did not report saccadic movements of visual targets but rather an extension of the pictures or a projection of the image he was looking at before the head movement on the one he was looking at after the movement. This overprojection faded after a few seconds.

His balance was improving with physical therapy allowing him to return to work, full time.

In May 2013, he presented an episode of “room tilt illusion” by pressing the tragus of the operated ear while sitting in a car at stopping. Suddenly he saw the car inverted of 180°. He clung to his seat under the dumbfounded look of the other passengers in the car. The episode lasted a few seconds (8). He came to our policlinic to be investigated.

**Figure 1:**
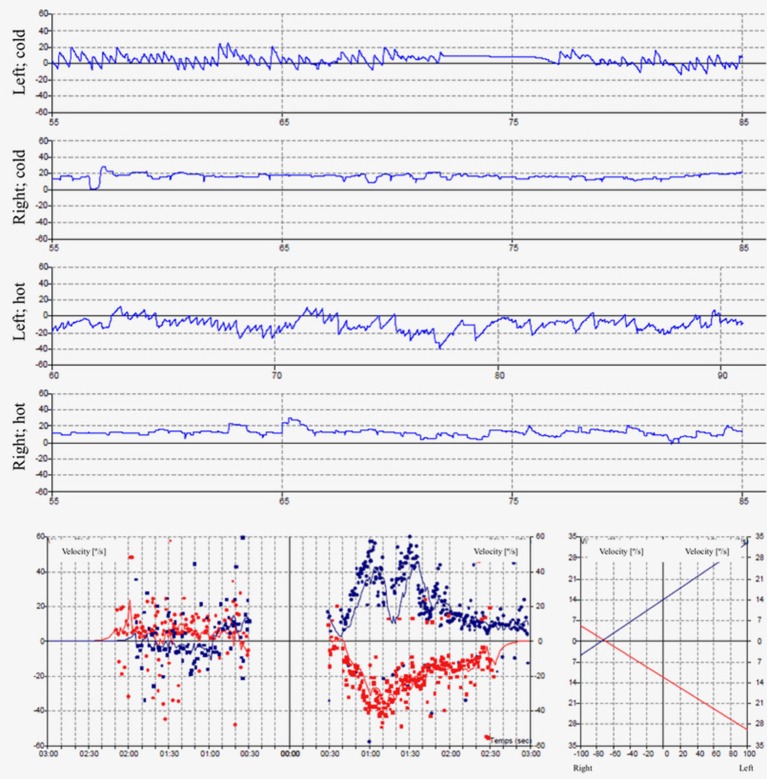
The caloric irrigations at 30 and 44° reveal a right areflexia with a peak slow phase velocity inferior to ≤ 5°/s.

**Figure 2:**
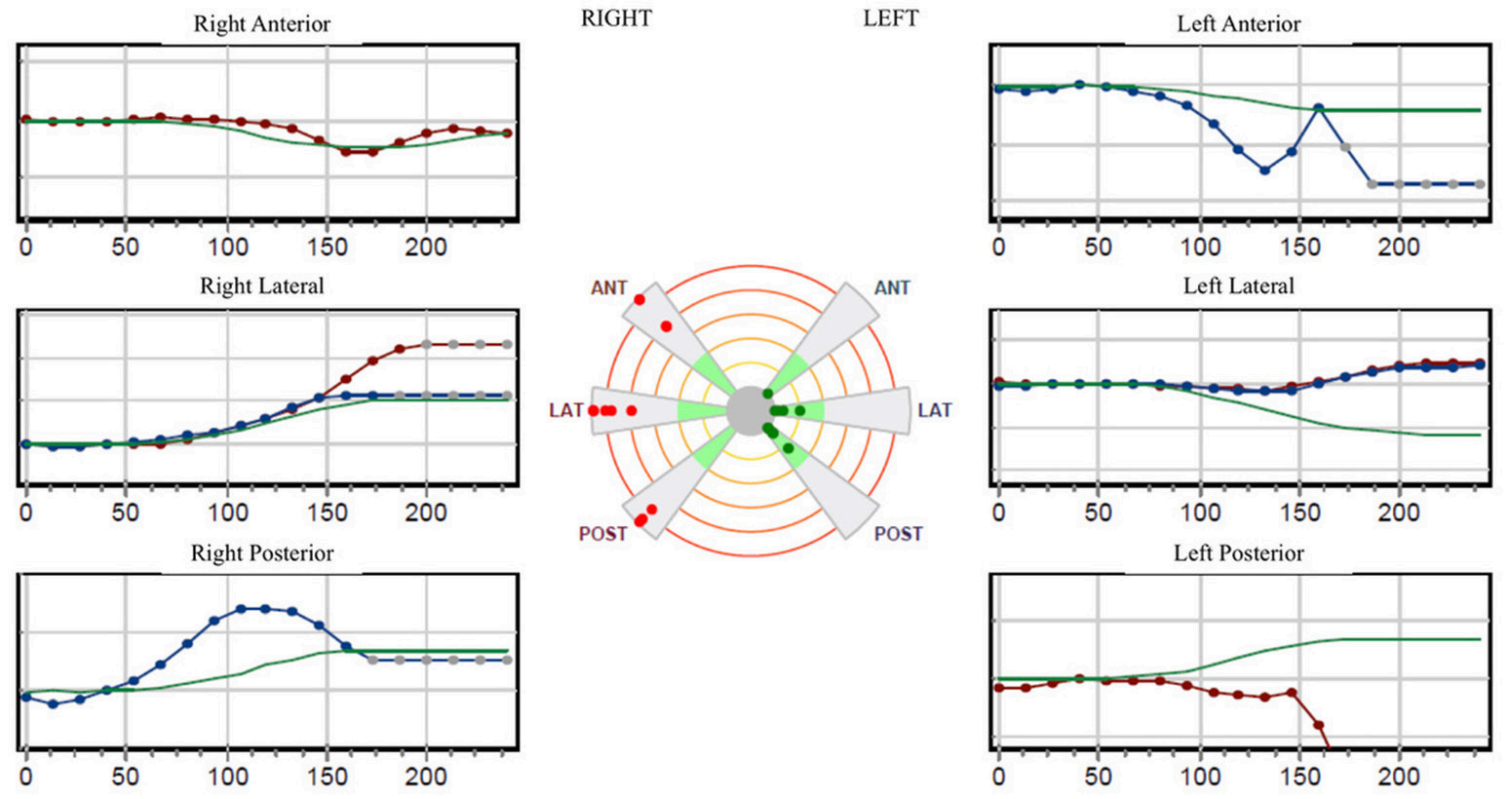
The video-head-impulse-test *(VHIT; Synapsis*^®^*; Marseille, F)* is pathological for the 3 right semicircular canals.

**Figure 3:**
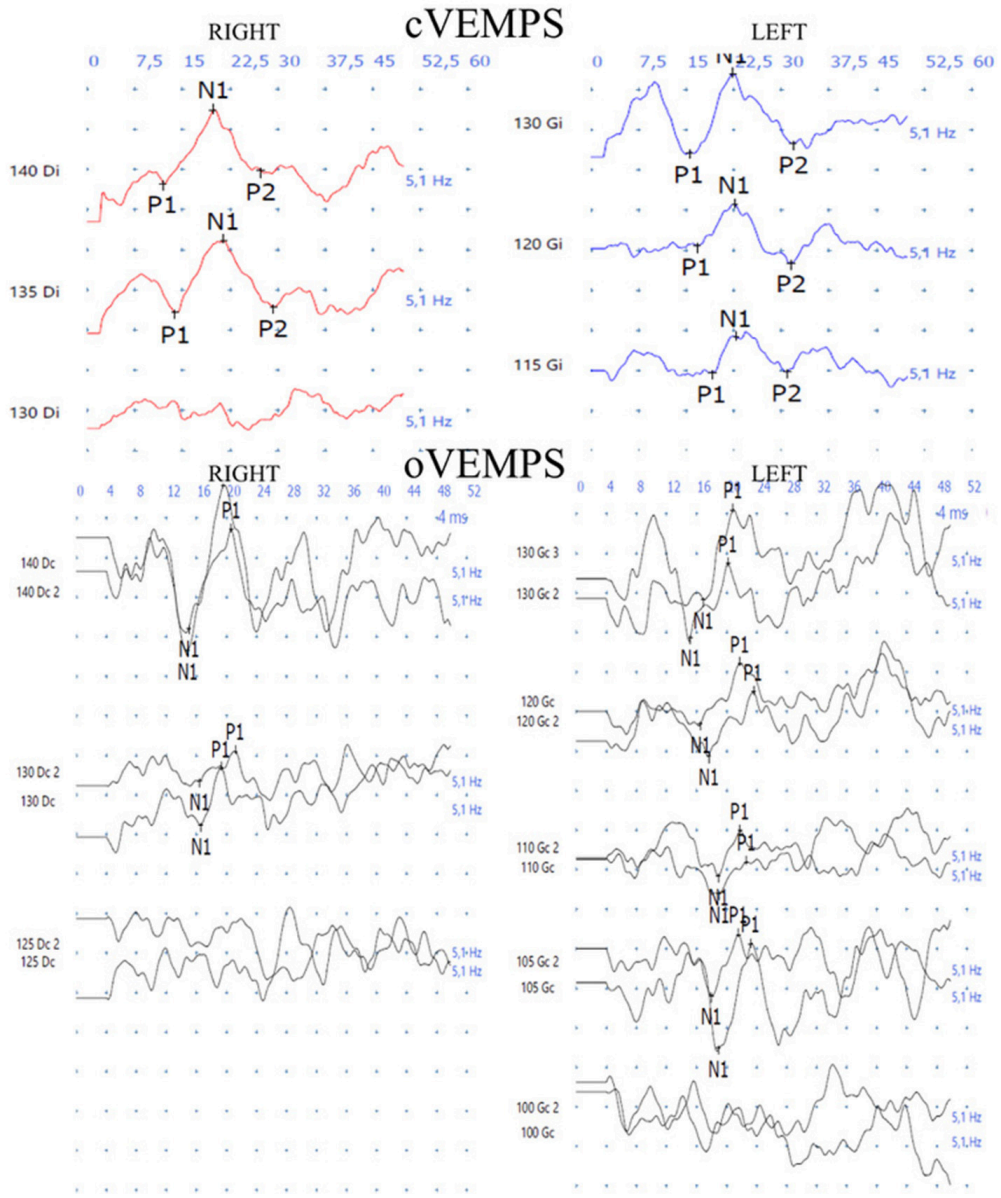
The cervical and ocular vestibular evoked myogenic potentials *(4 ms tone burst; 500 Hz; alternate polarity at 5 Hz)* are present on both sides.

The pure tone audiogram confirmed the known right deafness. The vestibular workup revealed a right areflexia to bithermal irrigations (peak slow phase velocity ≤ 5°/s at 30 and 44°; Figure 1), a head impulse test pathological for the 3 right semicircular canals (Figure 2), symmetrical responses at horizontal torsion swing tests (0.05–0.1 Hz, ωmax = 60°/s), and presence of cervical and ocular vestibular evoked myogenic potentials (Figure 3).

During the spring of 2015, he began to suffer from lumbar pain requiring regular intake of analgesics Dafalgan® (Paracetamol), Irfen® (Ibuprofene), and Tramal® (Tramadol), and infiltration at the level of L5 in August. He had to stop working as building painter.

**Figure 4:**
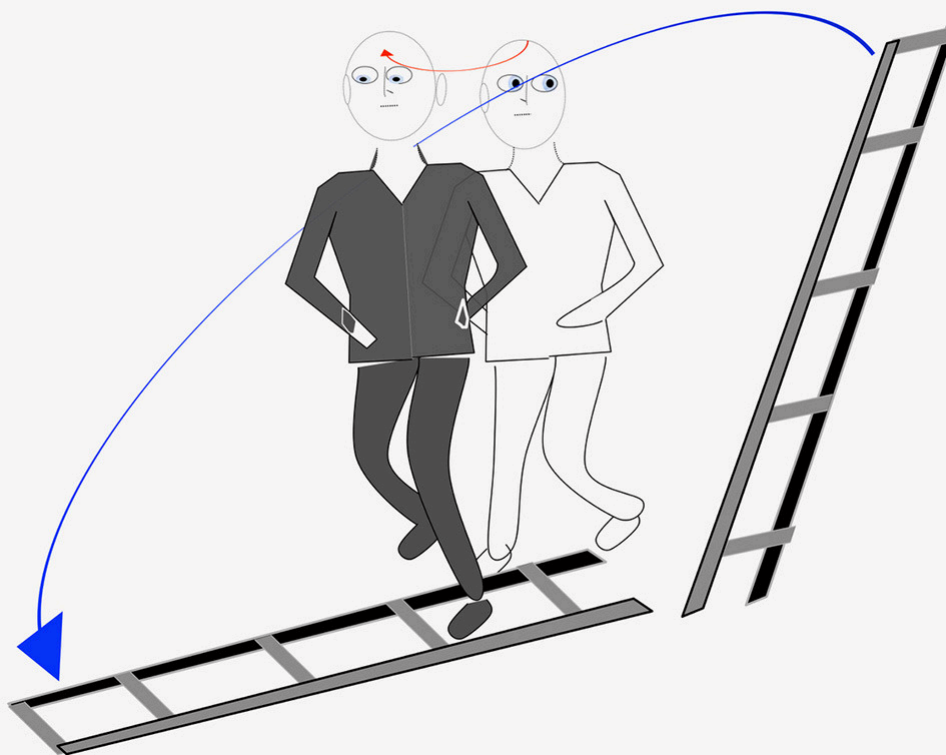
Graphic representation of the most impressive episode of palinopsia experienced by our patient.

In February 2016, the patient mentioned for the first time palinopsia phenomena after a particularly striking episode for him which occurred several months ago, before he needed medication for his lumbar pain. He hesitated to report it fearing to be considered crazy. While walking down the street along with his daughter, he looked on his left at a ladder leaning against the wall at the first floor of a building he had painted a few months earlier. When he looked again in front of him, he saw the ladder lying on the ground in front of him and began to walk in order to avoid putting his feet on the steps of the ladder. This ladder was of the same color and size as the one he had just seen against the wall on his left. When, after a few steps, his daughter told him that he was walking in a strange way, he became aware of his illusion (Figure 4). He had already presented several identical episodes, a little less clear and not having generated a momentary modification of his walking, and he did not consider it advisable to speak about it. The episodes always resulted of head rotations preferentially to the right but sometimes also to the left. He noticed that movement of the eyes only, without head movement, never triggered the phenomenon. The images were usually objects and rarely faces.

The neurologic examination showed no anomaly except some slight persistent instability to Romberg test. The visual acuity was 6/12 both sides and the visual field normal. An electroencephalogram and a cerebral MRI were normal.

## Discussion

The visual phenomenon reported by this patient corresponds to episodes of “immediate hallucinatory palinopsia.” Cases of palinopsia have been observed in patients suffering from focal cerebral lesions consecutive to trauma, parasites, abscesses, strokes, multiple sclerosis, tumors, arteriovenous malformations, particularly in cases involving the occipital and temporal lobes, affecting the non-dominant hemisphere. However, in 25% of cases palinopsia can result from lesions in the dominant hemisphere as in a woman suffering from a glioblastoma in the left inferior occipital gyrus (9). Palinopsia is usually accompanied by a visual field defect but some cases with a normal visual field have been observed such as that described by Praveen-Kumar and Rajesh (10), a patient affected by a temporo-occipital anaplastic oligodendrioglioma, or those of Ritsema et al. (11), one with an arteriovenous malformation in the lingual gyrus of the left occipital lobe and one with an ischemic stroke in the left peri-atrial region, near to the left optic radiations. In addition, multiple illicit drugs (marijuana, mescaline, lysergic acid dyethilamide, 3,4-methylenedioxymethamphetamine), and prescription drugs (interleukin 2, topiramate, trazodone, clomiphene citrate, nefazodone) can elicit episodes of palinopsia (12–14). Other known causes include poisoning with carbon monoxide or different other gazes, psychiatric conditions (Charles-Bonnet syndrome, schizophrenia, psychotic depression), metabolic and systemic diseases (non-ketotic hyperglycemia, diffuse cortical pathology), migraine, or diseases limited to the retina or optic nerve (3, 5). In some cases, no cause is identified (15).

None of these factors was identified in our patient. At the time of the most significant episode in which he saw a ladder on the floor after he saw it leaning against a wall, he was not taking any medication. It is therefore possible that, in his case, the episodes of palinopsia are linked to the right canalar vestibular deficit. His visual perception while performing the head impulse test, might be the expression of the first episodes of palinopsia. Indeed, while patients usually report an obvious jump of the visual image during rotations of the head toward the deficient side, our patient seems to transfer the visual target he was looking at before head rotation on the one that he should see at the end of the rotation.

The pathophysiology of palinopsia is not clear, but many hypothesis are proposed. One of them is that drugs could alter serotoninergic activity (12–14) or inhibit the synthesis of gamma-aminobutyric acid involved in the generation of action potentials of the visual system (16, 17). Another involves a local irritation of the occipito-parietal cortex by surrounding oedema (18). Finally, according to the Bayesian theory that says that surrounding images can be predicted using stored memories of previous images palinopsia could result from a mistake of the brain in the prediction phase of images (19).

In our patient we hypothesize that the phenomenon could be linked to alterations of peripheric vestibular function combined to a non-adaptation of the temporal binding process of visual and vestibular information. It has long been known that the otolithic and canalar functions converge and interact in the medial vestibular nucleus (20, 21) generally in the form of an inhibitory modulation of the canalar inputs by the otolithic ones (22, 23). More recently it has been shown that the velocity of the vestibular signal was slower than normal in case of deficient canalar function combined to a preserved otolithic function (24), like in our patient. It is also known that the vestibular system is faster than the visual system. Thus during a head rotation the vestibular inputs reach the cortex precociously while the visual inputs reach it later. This delay must be corrected by the brain by curbing the vestibular information so that the movement is perceived as unique by individual. If the temporal binding process of visual and vestibular information does not adapt to the slower than normal vestibular signals and continue to act normally, the vestibular inputs will remain be curbed and finally reach the cortex after the visual inputs. Thus, the patient will keep in mind the visual image that he had before head's movement and only will become aware of the new visual image once the information of movement given by the vestibular system arrives at the cortex.

Finally, it has been shown that the deficiency of the vestibulo-ocular reflex resulting from an alteration of canalar function combined to a preserved otolithic function worsens over time (24). This explains why the episodes of palinopsia only started with a vague sensation of projection of the image during the head impulse test to become evident only several months after the partial loss of the vestibular function.

To the best of our knowledge, no similar case has ever been reported. The literature mentions one patient who had a history of five ear operations for an aggressive cholesteatoma (25). This might have resulted in a vestibular deficit, at least partial, as a consequence of the numerous infectious episodes usually accompanying a cholesteatoma, or of an opening of the otic capsule by cholesteatoma or of the numerous surgeries. However, no mention is made about a partial vestibular loss and some factors existed in this patient who suffered from migraines and treated with topiramate which might be the cause of the episodes of palinopsia.

## Conclusion

In conclusion, this case illustrates that episodes of palinopsia may be due to disturbances of peripheral vestibular function. In patients for whom no previously known factor exists, we recommend a complete vestibular assessment including canalar and otolithic function tests.

## Author contributions

CS review of the literature, data collection about the patient, article writing, and editing. TL article editing. J-PG article writing and editing.

### Conflict of interest statement

The authors declare that the research was conducted in the absence of any commercial or financial relationships that could be construed as a potential conflict of interest.
